# Axon degeneration and PGC-1α-mediated protection in a zebrafish model of α-synuclein toxicity

**DOI:** 10.1242/dmm.013185

**Published:** 2014-03-13

**Authors:** Kelley C. O’Donnell, Aaron Lulla, Mark C. Stahl, Nickolas D. Wheat, Jeff M. Bronstein, Alvaro Sagasti

**Affiliations:** 1 Department of Molecular, Cell and Developmental Biology, University of California, Los Angeles, CA 90095, USA.; 2 Department of Neurology, David Geffen School of Medicine at UCLA, Los Angeles, CA 90095, USA.; 3 Parkinson’s Disease Research, Education, and Clinical Center, Greater Los Angeles Veterans Affairs Medical Center, Los Angeles, CA 90073, USA.

**Keywords:** PGC1α, Alpha synuclein, Axon, Mitochondria, Neurodegeneration, Zebrafish

## Abstract

α-synuclein (aSyn) expression is implicated in neurodegenerative processes, including Parkinson’s disease (PD) and dementia with Lewy bodies (DLB). In animal models of these diseases, axon pathology often precedes cell death, raising the question of whether aSyn has compartment-specific toxic effects that could require early and/or independent therapeutic intervention. The relevance of axonal pathology to degeneration can only be addressed through longitudinal, *in vivo* monitoring of different neuronal compartments. With current imaging methods, dopaminergic neurons do not readily lend themselves to such a task in any vertebrate system. We therefore expressed human wild-type aSyn in zebrafish peripheral sensory neurons, which project elaborate superficial axons that can be continuously imaged *in vivo*. Axonal outgrowth was normal in these neurons but, by 2 days post-fertilization (dpf), many aSyn-expressing axons became dystrophic, with focal varicosities or diffuse beading. Approximately 20% of aSyn-expressing cells died by 3 dpf. Time-lapse imaging revealed that focal axonal swelling, but not overt fragmentation, usually preceded cell death. Co-expressing aSyn with a mitochondrial reporter revealed deficits in mitochondrial transport and morphology even when axons appeared overtly normal. The axon-protective protein Wallerian degeneration slow (WldS) delayed axon degeneration but not cell death caused by aSyn. By contrast, the transcriptional coactivator PGC-1α, which has roles in the regulation of mitochondrial biogenesis and reactive-oxygen-species detoxification, abrogated aSyn toxicity in both the axon and the cell body. The rapid onset of axonal pathology in this system, and the relatively moderate degree of cell death, provide a new model for the study of aSyn toxicity and protection. Moreover, the accessibility of peripheral sensory axons will allow effects of aSyn to be studied in different neuronal compartments and might have utility in screening for novel disease-modifying compounds.

## INTRODUCTION

Parkinson’s disease (PD) is a movement disorder characterized pathologically by the loss of dopaminergic cells in the midbrain, and by the appearance of Lewy bodies (Braak et al., 1999; Braak et al., 2003), which are intracellular protein aggregates composed primarily of ubiquitin and α-synuclein (aSyn) (Spillantini et al., 1997; Spillantini et al., 1998). *SNCA*, the gene that encodes aSyn, was the first gene to be associated with PD: duplications, triplications and mutations in this gene are associated with rare hereditary forms of the disease (Polymeropoulos et al., 1997; Krüger et al., 1998; Singleton et al., 2003; Fuchs et al., 2007), and variants are also associated with the more common sporadic form of PD (Satake et al., 2009; Simón-Sánchez et al., 2009; Wu-Chou et al., 2013). aSyn is a synaptic protein (Maroteaux et al., 1988; Boassa et al., 2013). Aggregate formation in the synapse and axon precedes Lewy body formation and cell death in multiple cell types (Galvin et al., 1999; Orimo et al., 2008; Schulz-Schaeffer, 2010; Nakata et al., 2012). These recent findings have led to the hypothesis that PD degeneration is initiated in the axon (O’Malley, 2010; Burke and O’Malley, 2012). Whether axon degeneration leads to cell death or proceeds independently, however, is unknown.

A number of lines of evidence support the hypothesis that mitochondrial dysfunction contributes to PD pathogenesis. Mitochondrial dysfunction has been observed in postmortem samples from individuals with PD (Schapira et al., 1990; Penn et al., 1995; Navarro et al., 2009), and a number of genes associated with mitochondrial function are associated with hereditary forms of the disease (Martin, 2006; Dodson and Guo, 2007; Sai et al., 2012). Although aSyn itself is not a mitochondrial protein, it is capable of binding mitochondria directly (Nakamura et al., 2011) and can accumulate on the inner and outer mitochondrial membranes (Li et al., 2007; Zhu et al., 2012). Its overexpression or mutation alters mitochondrial morphology in a number of systems and cell types (Martin et al., 2006; Li et al., 2007; Kamp et al., 2010; Nakamura et al., 2011; Xie and Chung, 2012; Zhu et al., 2012), and is associated with respiratory chain defects, oxidative stress and mitochondrial fragmentation (Parihar et al., 2008; Chinta et al., 2010; Zhu et al., 2012). A better understanding of the effect of aSyn on mitochondrial transport and function *in vivo* could provide insight into PD pathophysiology and potential therapeutic targets.

Each of the models used to study aSyn-induced degeneration has advantages and limitations. *In vitro* studies can shed light on the cell biology of aSyn oligomerization and aggregation, but their relevance to pathophysiology in living animals is unknown. By contrast, studies in mammalian systems recapitulate some disease phenotypes, but *in vivo* cell biological studies are difficult (Martin et al., 2006; Chesselet, 2008). A better understanding of aSyn toxicity requires a model system in which neurons can be visualized and manipulated *in vivo*. Larval zebrafish are increasingly recognized as being a genetically and pharmacologically tractable model system useful in high-throughput screens for PD-associated phenotypes (Bretaud et al., 2004; Flinn et al., 2008). Moreover, their optical transparency permits the visualization of cellular processes in living animals, including mitochondrial transport (Plucińska et al., 2012). The zebrafish model could therefore prove to be a useful tool for studying the relationship between aSyn expression and neurodegeneration at the cellular level.

**TRANSLATIONAL IMPACT**

**Clinical issue**

Accumulation of the neuronal protein α-synuclein (aSyn) is associated with multiple neurodegenerative disease processes, including Parkinson’s disease. The sequence of subcellular events that underlie neurodegeneration in these diseases is not well understood. However, recent studies suggest that axon pathology might arise early in pathogenesis and might lead to the functional deficits that precede and eventually lead to cell loss. Attempts to preserve neuronal circuitry and prevent neurodegeneration therefore require a better understanding of the mechanisms by which axons degenerate, which can only be achieved through longitudinal, *in vivo* monitoring of different neuronal compartments.

**Results**

In this study, the authors establish an *in vivo* model for longitudinally studying the effects of aSyn accumulation on axonal integrity by expressing human wild-type aSyn in zebrafish peripheral sensory neurons, which are accessible to imaging in living animals. They report that the expression of aSyn induces cell death in peripheral sensory neurons but that axon pathology occurs earlier and more frequently than cell death. Time-lapse imaging reveals that axonal fragmentation does not consistently proceed in a retrograde direction from the axon terminal to the cell body. The authors then use *in vivo* imaging of axonal mitochondria to reveal early defects in mitochondrial morphology and transport, and eventual accumulation of the organelles in axonal varicosities. Notably, the axon-protective protein Wallerian degeneration slow (WldS) delays the onset of axonopathy in the zebrafish model but does not affect cell death or axonal fragmentation, whereas overexpression of PGC-1α, which has roles in mitochondrial biogenesis and reactive-oxygen-species scavenging, provides robust protection against both axon pathology and cell death.

**Implications and future directions**

These results suggest that axonopathy is an early consequence of aSyn accumulation, which only sometimes leads to cell death. They also suggest that mitochondrial impairment might be relevant to the pathophysiology of neurodegenerative diseases that involve aSyn accumulation, and that PGC-1α-mediated protection could be a promising therapeutic target. More generally, because the axonal compartment is especially sensitive to disruptions in mitochondrial function and transport, a better understanding of the relationship between mitochondrial function and axonal integrity could identify new therapeutic targets that act on pathways either upstream of or parallel to cell death. Further use of the model system established here might therefore yield new insights into the vulnerability of the axonal compartment to aSyn toxicity, and into the relationship between axon degeneration and cell death in neurodegenerative diseases.

We expressed human aSyn in zebrafish Rohon-Beard neurons, peripheral sensory neurons in the developing spinal cord that project sensory axons to the skin. Both the cell bodies and the elaborate peripheral arbors of these cells can be monitored *in vivo*, permitting visualization of axonal transport and degeneration (Plucińska et al., 2012). Co-expressing aSyn and GFP resulted in moderate cell death, and many axons exhibited diffuse or focal swellings associated with degeneration of this compartment. Expression of the axon-protective protein Wallerian degeneration slow (WldS) (Lunn et al., 1989; Coleman et al., 1998) delayed axon degeneration, but did not affect cell death. Early defects in mitochondrial morphology and transport suggested that mitochondrial toxicity might be relevant to this observed pathogenesis. Consistent with this hypothesis, expression of PGC-1α, a transcriptional coactivator with roles in mitochondrial biogenesis and reactive oxygen species (ROS) detoxification, prevented both axonopathy and cell death caused by aSyn.

## RESULTS

**Fig. 1:**
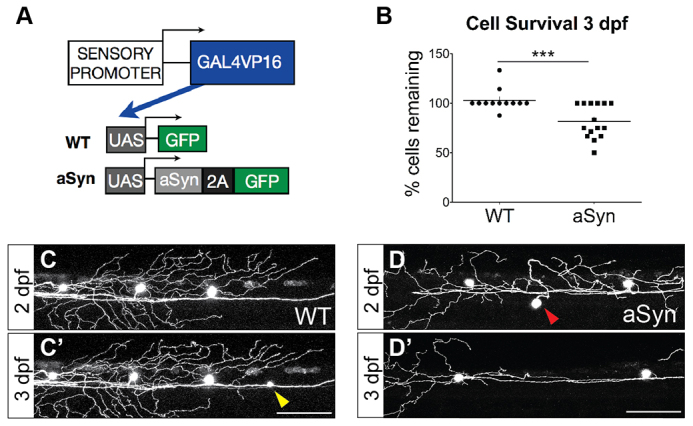
**Alpha-synuclein is moderately toxic to zebrafish sensory neurons between 2 and 3 dpf.** (A) Transgenes to express GFP (WT) or aSyn-2A-GFP (aSyn) were injected into wild-type embryos at the one-cell stage. The *CREST3* enhancer drove expression in peripheral sensory neurons. The Gal4-UAS system was used to amplify gene expression, and a viral 2A sequence was cloned between aSyn and GFP to generate two proteins from a single transcript. (B-D) Approximately 20% of aSyn-expressing neurons died between 2 and 3 days post-fertilization (dpf) (WT 3-dpf survival: 102.9±3.2%; aSyn: 81.7±4.4%; *n*≥12 embryos, ***P=0.0010). Some cells newly expressed GFP during the imaging period (yellow arrowhead in C). Red arrowhead in D points to a cell that died between 2 and 3 dpf. Scale bars: 100 μm.

Zebrafish Rohon-Beard neurons in the spinal cord arborize in the skin, making them readily accessible to *in vivo* imaging of dynamic intracellular processes. We generated transgenes to overexpress aSyn in these cells, using a sensory-neuron promoter and the Gal4-UAS binary transcription system to drive robust gene expression (Fig. 1). To co-express aSyn and GFP, we used the viral 2A system (Donnelly et al., 2001), which provides bright reporter expression earlier than the aSyn-2A-DsRed transgene previously reported (Prabhudesai et al., 2012). The viral 2A system permits visualization of cells expressing the transgene, but circumvents the possibility of increased aggregation that could potentially be observed with a fusion protein. Consistent with a previous report (Prabhudesai et al., 2012), immunostaining for human aSyn revealed protein expression and aggregate formation by 2 days post-fertilization (dpf) in aSyn-injected cells, but not in control cells expressing GFP alone (supplementary material Fig. S1).

When the HuC promoter is used to drive aSyn expression in larval zebrafish neurons, embryos exhibit massive cell death and gross morphological abnormalities, and die within 2-3 dpf (Prabhudesai et al., 2012). When we drove expression using a sensory-neuron promoter, only a small number of embryos exhibited such defects; most were morphologically normal. Only the latter were retained for subsequent studies, and in these embryos lethality was not observed at levels higher than in wild type.

### Alpha-synuclein causes moderate cell death in larval zebrafish sensory neurons

To determine whether aSyn caused early toxicity in sensory neurons, we injected the aSyn-2A-GFP construct into transgenic embryos from a stable line expressing DsRed in sensory neurons, and screened for reporter expression at 1 dpf. Cells were imaged hourly between 32 and 44 hours post-fertilization (hpf) (supplementary material Fig. S2A,B). Because transient aSyn-2A-GFP expression was sparse, some neurons expressed only DsRed; these served as an internal control for development and cell death. Over the course of the imaging period, peripheral sensory axons extended normally in aSyn-expressing neurons (supplementary material Fig. S2B), and cell survival between the first and last time point was not different between the two groups (supplementary material Fig. S2C). These observations indicate that aSyn is not toxic at early stages.

Having determined that aSyn expression does not impair development of peripheral sensory neurons by 44 hpf, we investigated whether it affected cell survival at later time points (Fig. 1B–D). Cohorts of embryos expressing GFP (WT) or aSyn-2A-GFP were monitored between 2 and 3 dpf, and Rohon-Beard neurons were counted at each time point (Fig. 1B–D). Approximately 20% of cells in aSyn-expressing embryos died between 2 and 3 dpf (Fig. 1B; WT 3-dpf survival: 102.9±3.2%; aSyn: 81.7±4.4%; *n*≥12 embryos, *P*=0.0010).

### Alpha-synuclein expression causes axonopathy

**Fig. 2:**
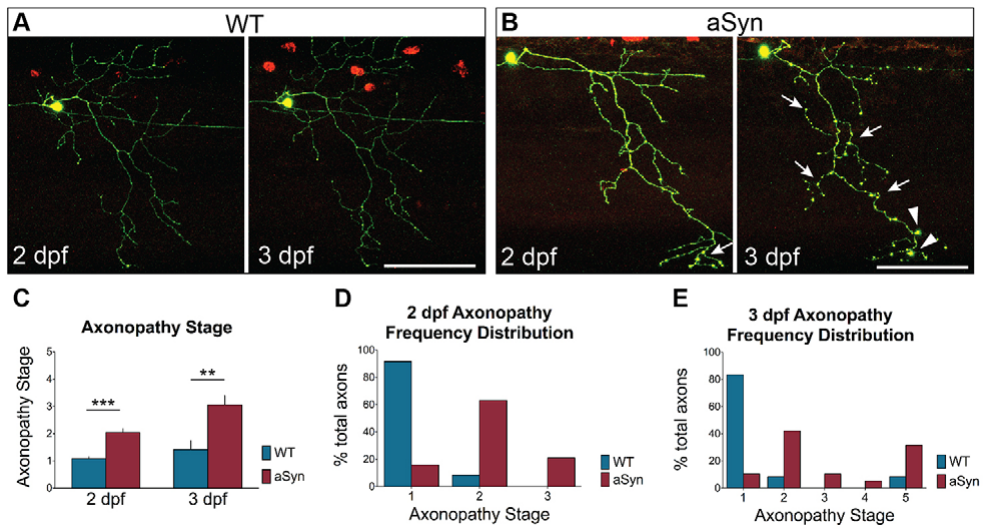
**Early axonopathy in aSyn-expressing peripheral sensory neurons.** Axon pathology was scored at 2 and 3 days post-fertilization (dpf) in WT and aSyn-expressing axons, using the 5-point staging system described in supplementary material Fig. S3. (A) At 2 dpf (48-58 hpf) and 3 dpf (72-82 hpf), WT axons were smooth and continuous (score of 1). (B) At 2 dpf, axonal beading (arrows) was observed in many aSyn-expressing cells. By 3 dpf, axonal dystrophy in aSyn-expressing cells was more severe, with more diffuse beading (arrows) and larger varicosities (arrowheads). (C) Quantification of average axonopathy stage in wild-type and aSyn-expressing embryos. aSyn-expressing axons were more dystrophic at both 2 dpf (WT degeneration stage: 1.03±0.08, *n*=12 axons in 5 animals; aSyn: 2.05±0.14; *n*=19 axons in 8 animals; ***P<0.0001) and 3 dpf (WT: 1.42±0.34; aSyn: 3.05±0.35; *n*≥12 axons in ≥5 animals as above; **P=0.0033). (D,E) Histograms representing frequency distribution of axonopathy stage. At 2 dpf (D), 11/12 wild-type axons (91.7%) were smooth and continuous (stage 1); one exhibited mild beading (stage 2). By contrast, only 3/19 (15.8%) aSyn-expressing axons were at stage 1; 12/19 (63.2%) exhibited mild beading (stage 2), and 4/19 (21.1%) exhibited more severe axonopathy (stage 3). (E) Frequency histogram of axonopathy distribution in the same cells at 3 dpf. One wild-type axon (8.3%) exhibited mild beading (stage 2), and one wild-type cell had undergone developmental cell death (stage 5). All remaining wild-type axons (10/12, 83.3%) were smooth and continuous (stage 1). By contrast, only 2/19 (10.5%) aSyn-expressing axons remained at stage 1 by 3 dpf. 6/19 (31.6%) had fully degenerated (stage 5), and the remaining 11/19 (57.9%) were in intermediate stages of degeneration (8/19 in stage 2; 2/19 in stage 3; 1/19 in stage 4). Scale bars: 100 μm.

Axon pathology is often characterized by swelling or beading in the axon that might precede fragmentation (Beirowski et al., 2010; Nikić et al., 2011). To quantify axonal dystrophy in cells expressing DsRed and either GFP or aSyn-2A-GFP at 2 and 3 dpf (Fig. 2A), we developed a 5-point staging system (supplementary material Fig. S3). At 2 dpf, before cell death had been observed, the majority (14/19, 73.7%) of aSyn-expressing axons exhibited a beaded morphology, quantified as degeneration stage 2-3 (Fig. 2; WT degeneration stage: 1.08±0.08; aSyn: 2.05±0.14; *n*≥12 axons, *P*<0.0001). When the same axons were imaged the following day, degeneration was further advanced (Fig. 2B; WT degeneration stage: 1.42±0.33; aSyn: 3.05±0.35; *n*≥12 axons, *P*=0.0033). One control axon died between 2 and 3 dpf (degeneration stage 5), and one exhibited mild beading (stage 2). The remaining ten control axons were smooth and continuous (stage 1). Among aSyn-expressing axons, by contrast, 17/19 axons (89.5%) received a degeneration score of 2 or higher, with six degenerating entirely (stage 5).

### Axonopathy, but not axonal fragmentation, precedes cell death in aSyn-expressing cells

**Fig. 3:**
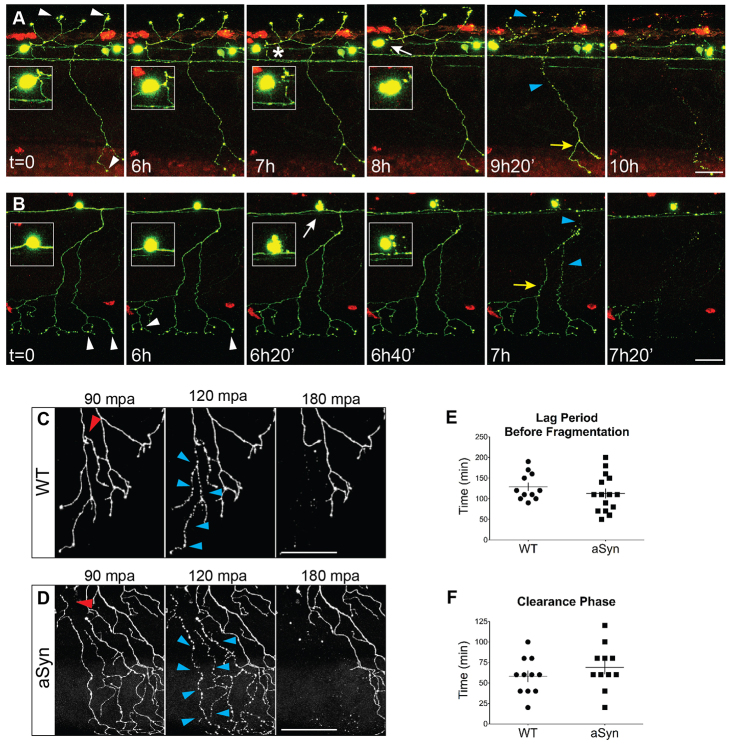
**Axonopathy is not followed by ‘dying back’ or Wallerian-like degeneration in aSyn-expressing neurons.** (A,B) Time-lapse imaging of neurodegeneration. Cells were imaged every 20 minutes beginning 54 hours post-fertilization (hpf). Axons from at least 11 embryos from each group were transected; representative images from aSyn-expressing animals are shown. Time stamps in images are relative to the start of the imaging period. Axonal varicosities were observed (white arrowheads) several hours before cell death. White arrows point to morphological changes indicative of cell death. Inset represents cell body magnified 2×. Asterisk in A indicates separation of the axon from the cell body. Axonal fragmentation (blue arrowheads) usually did not occur before cell death, and was not stereotyped: it did not occur synchronously along the length of the axon, nor in a retrograde direction (yellow arrows point to distal portions of the axon that are still intact). (C,D) Representative images of wild-type (C) and aSyn-expressing (D) axons undergoing WD after transection with a two-photon laser. Axons were transected with a two-photon laser at 2 dpf, and embryos were imaged every 30 minutes for up to 12 hours. Red arrowhead points to site of transection. After injury, in both wild-type and aSyn-expressing axons, fragmentation was synchronous along the length of the transected axon (blue arrowheads). mpa, minutes post-axotomy. (E) There was no difference in the duration of the lag period between transection and fragmentation (WT: 129.1±10.0 minutes, *n=*11 axons from 11 animals; aSyn: 112.7±11.7 minutes; *n*=15 axons from 15 animals, *P*=0.3173). (F) The time between fragmentation and clearance of all axonal debris was not significantly different between the two groups (WT: 58.2±6.9 minutes; aSyn: 69.1±8.3 minutes; *P*=0.3213). Scale bars: 50 μm.

It has recently been proposed that the axon degeneration observed in PD represents an early, and potentially independent, process in pathophysiology (O’Malley, 2010; Burke and O’Malley, 2012; Jellinger, 2012). In zebrafish neurons expressing aSyn, the percentage of cells with dystrophic axons between 2 and 3 dpf was higher than the percentage of cells that died during that period. To determine whether severe axonopathy always preceded cell death, we conducted time-lapse imaging at 20-minute intervals between 56 and 68 hpf (Fig. 3A,B). In cells that died during the imaging period, the onset of axonal dystrophy (beading or fragmentation) was compared with morphological changes in the soma that herald cell death. In all cases (*n=*9), focal or diffuse swellings (axonopathy stage 2-3) were seen in axons several hours before cell death (Fig. 3A,B). Axonal fragmentation, however, did not precede apoptotic changes in the cell body (Fig. 3A,B, arrows). Overt axonal breakdown therefore does not proceed directly to the death of the cell body in this model. However, because axonal dystrophy preceded cell death, it is likely that the axonal compartment is more vulnerable to aSyn toxicity.

### Axonal injury increases cell death in aSyn-expressing neurons

To further investigate the sensitivity of the axon and cell body to aSyn toxicity, we examined the effect of aSyn expression on the rate of Wallerian degeneration (WD) after injury. WD is the process by which severed axons degenerate after separation from the cell body. In most neuronal populations, including zebrafish peripheral sensory neurons (Martin et al., 2010), WD after axonal transection is compartment-specific: the distal fragment degenerates, whereas the proximal axon and cell body survive. To determine whether aSyn expression alters these characteristics, we transected axons at 2 dpf and conducted time-lapse confocal imaging to visualize WD *in vivo* (Fig. 3C,D). aSyn expression did not change the duration of the lag phase before fragmentation (Fig. 3E), or the clearance of axonal debris (Fig. 3F). WD in aSyn-expressing axons therefore proceeds with the same rapid and stereotyped kinetics as in wild-type axons. In aSyn axons, as in wild type, fragmentation of the distal axon was synchronous (Fig. 3C,D), unlike the axon degeneration observed in uninjured aSyn-expressing cells (Fig. 3A,B).

Consistent with the compartment specificity of WD, in both wild-type and aSyn-expressing axons the cell body and proximal axon remained intact, whereas the distal fragment underwent degeneration (data not shown). However, when we imaged transected cells at 3 dpf, 24 hours after injury, 50% of aSyn-expressing cells (5/10) had died, whereas all axotomized WT cells (*n*=11) were still intact. Because 20% of uninjured aSyn-expressing cells died between 2 and 3 dpf (Fig. 1B), this higher percentage suggests that direct axonal injury exacerbates aSyn toxicity.

### WldS delays axon degeneration caused by aSyn toxicity

**Fig. 4:**
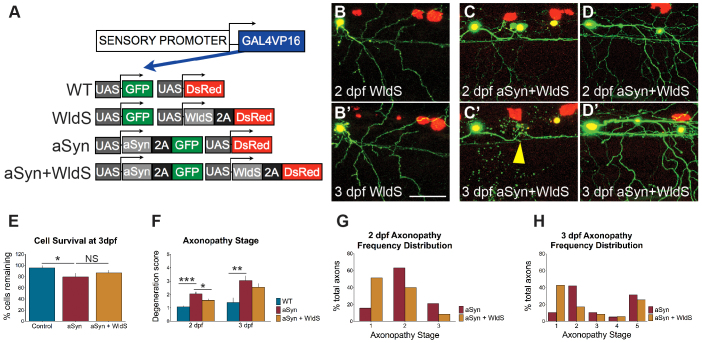
**WldS delays axonopathy but does not prevent cell death caused by aSyn toxicity.** (A) Transgenes used to visualize the effect of aSyn and WldS expression on peripheral sensory neurons. (B) Representative images of WldS-expressing control cells at 2 (B) and 3 (B′) days post-fertilization (dpf). Axons were smooth and continuous. (C,D) Representative images of cells expressing both aSyn and WldS. At 2 dpf, aSyn+WldS-expressing axons were on average more continuous (compare with aSyn in Fig. 2). WldS did not prevent degeneration of axons in cells that died between 2 and 3 dpf (C,C′; yellow arrowhead points to degenerated soma). Axons that remained connected to cell bodies were relatively preserved (D,D′). (E) WldS did not affect survival of aSyn-expressing cells between 2 and 3 dpf (WT: 95.65±4.35%; aSyn: 79.36±6.62%; WldS+aSyn: 86.86±4.43%; *n*=22 animals per group; *P=0.3515). (F) Average axonopathy stage at 2 and 3 dpf. WldS-expressing aSyn axons were significantly protected at 2 dpf (aSyn: 2.05±0.14, *n*=19 axons from 8 animals; WldS+aSyn: 1.57±0.11; *n*=35 axons from 11 animals, *P=0.0114). By 3 dpf, this difference was no longer significant (aSyn: 3.05±0.35; WldS+aSyn: 2.54±0.28; *P*=0.2766). ***P<0.0001; **P=0.0033. (G,H) Frequency distribution of axonopathy scores at 2 (G) and 3 (H) dpf. At 3 dpf, axons that underwent cell death (aSyn: 6/19, 31.6%; aSyn+WldS: 9/35, 25.7%) had fully degenerated (axonopathy stage 5), regardless of whether or not WldS was expressed. Wild-type and aSyn axonopathy data were replotted from Fig. 2. Scale bar: 50 μm.

To further characterize aSyn-induced degeneration, we sought to determine whether it could be prevented by the axon-protective protein WldS (Fig. 4). This protein was first discovered to delay WD of transected axons (Coleman et al., 1998; Mack et al., 2001) and subsequently found to be protective of axons in many animal models of neurodegenerative disease (Sajadi et al., 2004; Hasbani and O’Malley, 2006; Press and Milbrandt, 2008; Cheng and Burke, 2010). aSyn and WldS were co-expressed in peripheral sensory neurons (Fig. 4A,C,D), and cell survival and axon pathology were quantified between 2 and 3 dpf (Fig. 4C–H). WldS did not significantly protect against cell death induced by aSyn (Fig. 4E). Axon degeneration, however, was delayed in WldS-expressing cells (Fig. 4F–H). Degeneration scores were lower at 2 dpf in WldS-expressing cells but, by 3 dpf, this difference was no longer significant (Fig. 4F). In cells that died between 2 and 3 dpf, WldS had no axon-protective effect (stage 5; Fig. 4C,H). However, a higher percentage of cells expressing WldS had healthy (stage 1) axons at both 2 dpf (Fig. 4G) and 3 dpf (Fig. 4H) than cells expressing aSyn alone. WldS therefore provided moderate protection against aSyn toxicity in the axonal compartment, reducing the incidence of focal swellings in axons connected to intact cell bodies. However, WldS could delay neither aSyn-induced cell death nor the axon degeneration associated with it.

### Mitochondrial pathology in axons of aSyn-expressing neurons

**Fig. 5:**
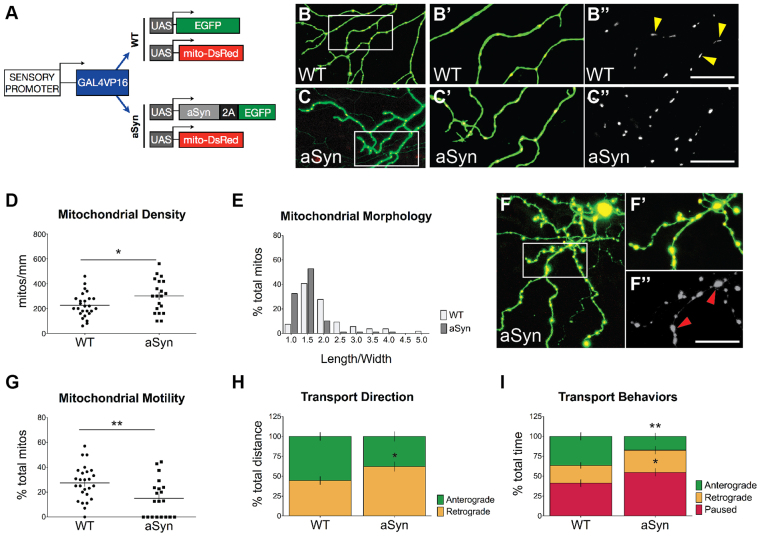
**Early mitochondrial pathology and transport impairments in aSyn-expressing axons.** (A) Transgenes used to visualize mitochondria in GFP-(WT) or aSyn-2A-GFP-expressing peripheral sensory neurons. Transgenes were co-injected into wild-type embryos at the one-cell stage. WT (B-B″) and aSyn-expressing (C-C″) cells were imaged at 2 dpf. Yellow arrowheads point to elongated mitochondria in wild-type axons. (D) Mitochondrial density was higher in aSyn-expressing cells (WT: 226.2±19.1 mitochondria/μm, *n*=26 axons in 12 animals; aSyn: 302.0±29.8 mitochondria/μm; *n*=20 axons in 10 animals, *P =0.031). (E) Mitochondrial morphology was quantified as the ratio of length to width in individual mitochondria. Values were binned and the frequency distribution was plotted on a histogram. Mitochondria in aSyn-expressing axons were more spherical than in wild-type axons, with fewer mitochondria exhibiting a high length:width ratio. (F) Large, swollen mitochondria occupied the spheroids in dystrophic aSyn-expressing axons. Boxed region in F is represented in F′-F″. Red arrowheads point to enlarged mitochondria. [Note that the scale bar in F″ is the same as in B″ and C″ (100 μm).] (G) Mitochondrial transport was evaluated along 50-μm axonal segments every second. Overall mitochondrial transport was significantly reduced in aSyn-expressing axons (WT % motile: 27.4±2.7%; aSyn: 15.05±3.4%; *n*≥20 axons in ≥10 animals per group, as above; **P =0.0061). (H) A higher percentage of distance traveled by motile mitochondria was in the retrograde direction (WT % retrograde distance: 44.61±5.07%; aSyn: 62.17±6.06%; *n*≥52 mitochondria; *P =0.0300). (I) Motile mitochondria spent less time moving in the anterograde direction (WT: 36.44±4.80%; aSyn: 17.39±4.05%; *n*≥52 mitochondria; **P=0.0063), and a greater percentage of time paused than in wild-type axons (WT: 41.25±4.43%; aSyn: 54.82±4.85%; *n*≥52 mitochondria; *P=0.0478).

Multiple *in vitro* and histological studies suggest that both wild-type and mutant aSyn interact with mitochondria (Martin et al., 2006; Parihar et al., 2008; Banerjee et al., 2010; Chinta et al., 2010; Devi and Anandatheerthavarada, 2010; Nakamura et al., 2011; Calì et al., 2012; Reeve et al., 2012; Zhu et al., 2012). To determine whether axonal mitochondria were affected by aSyn expression in our model, DsRed fused to the cox8 mitochondrial matrix targeting signal was co-expressed in sensory neurons with either GFP or aSyn-2A-GFP (Fig. 5A–C). Mitochondrial density was significantly higher in aSyn-expressing cells, even in the absence of overt axonopathy (Fig. 5C,D). Mitochondria in aSyn-expressing axons were less elongated than in wild-type cells (Fig. 5C,E; WT length/width: 2.01±0.11; aSyn: 1.48±0.05; *n*≥54 mitochondria from ≥5 embryos; *P*<0.0001), with a higher percentage of spherical mitochondria (ratio of 1), a phenotype associated with respiratory chain dysfunction (Benard and Rossignol, 2008). In dystrophic aSyn-expressing axons (Fig. 5F), many mitochondria exhibited pathological swelling characteristic of the mitochondrial permeability transition (Haworth and Hunter, 1979; Kowaltowski et al., 1996; Brustovetsky et al., 2002).

Because mitochondrial transport arrest is associated with axon degeneration (Baloh et al., 2007; Kim-Han et al., 2011; Sterky et al., 2011; Avery et al., 2012), we investigated whether aSyn expression induced mitochondrial transport impairments at 2 dpf, prior to axonal fragmentation and cell death. Mitochondrial transport was evaluated along 50-μm axonal segments for 6 minutes in wild-type or aSyn-expressing sensory neurons. Kymographs were generated to quantify overall motility, defined as the percentage of mitochondria that moved within a 6-minute time-lapse movie. Mitochondrial motility was significantly reduced in aSyn-expressing axons (Fig. 5G). A higher percentage of the total distance traveled by the remaining motile mitochondria was in the retrograde direction (Fig. 5H). Motile mitochondria spent less time moving in the anterograde direction, and a greater percentage of time paused than mitochondria in wild-type axons (Fig. 5I). The speed of uninterrupted runs in either the anterograde or retrograde direction, however, was not significantly different between wild-type and aSyn-expressing cells (WT anterograde speed: 0.56±0.04 μm/s; aSyn: 0.53±0.06 μm/s; *n*≥27 mitochondria, *P*=0.7137; WT retrograde speed: 0.57±0.04 μm/s; aSyn: 0.64±0.07 um/s; *n*≥38 mitochondria from ≥10 embryos). Early mitochondrial pathology in aSyn-expressing axons might therefore contribute to degeneration in this model.

### PGC-1α expression mitigates toxicity in aSyn-expressing sensory neurons

**Fig. 6:**
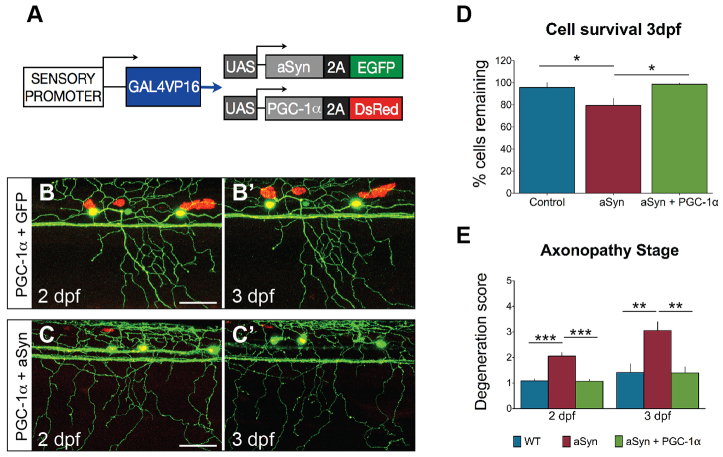
**PGC-1α mitigates aSyn toxicity.** (A) Transgenes co-injected to express PGC-1α and aSyn in sensory neurons. (B,C) Cells were imaged between 2 and 3 dpf. (D) PGC-1α prevented cell death in aSyn-expressing cells at 3 dpf (aSyn: 79.36±6.62%; aSyn+PGC-1α: 98.61±0.95%; *n*≥18; *P =0.0129). (E) PGC-1α prevented axonopathy at 2 dpf (aSyn degeneration score: 2.05±0.14; aSyn+PGC-1α: 1.07±0.07; *n*≥15; ***P<0.0001). At 3 dpf, PGC-1α-expressing axons were still protected at a level equivalent to controls (WT degeneration score: 1.42±0.34, *n*=12 axons in 6 animals; aSyn: 3.05±0.35, *n*=19 axons in 8 animals; aSyn+PGC-1α: 1.40±0.22; *n*=10 axons in 5 animals; one-way ANOVA with Newman-Keuls post-test, **P=0.0052). Scale bars: 50 μm. Wild-type and aSyn data were replotted from Fig. 2.

Because mitochondrial defects appeared early in aSyn-expressing axons, we hypothesized that mitochondrial dysfunction was directly involved in degeneration. To investigate whether improved mitochondrial function could prevent degeneration in aSyn-expressing sensory neurons, the transcriptional coactivator PGC-1α was expressed in these cells. PGC-1α plays a number of regulatory roles in mitochondrial biogenesis and ROS detoxification (Wu et al., 1999; St-Pierre et al., 2006), and PGC-1α overexpression is protective in multiple models of neurodegeneration (St-Pierre et al., 2006; Keeney et al., 2009; Shin et al., 2011; Mudò et al., 2012). We have documented that PGC-1α expression in zebrafish peripheral sensory neurons increases mitochondrial volume and density, and prevents injury-induced changes in mitochondrial redox homeostasis (O’Donnell et al., 2013). Co-expressing PGC-1α and aSyn in peripheral sensory neurons (Fig. 6A) robustly protected against aSyn toxicity between 2 and 3 dpf (Fig. 6B–E). Unlike WldS, PGC-1α reversed both cell death (Fig. 6D) and axonopathy (Fig. 6E) in aSyn-expressing cells. These results are consistent with mitochondrial dysfunction playing a key role in aSyn-induced toxicity.

## DISCUSSION

aSyn accumulation is associated with neurodegeneration, but the cellular mechanisms that underlie its toxicity are not well understood. We have expressed human wild-type aSyn in zebrafish peripheral sensory neurons, and observed aggregate formation and moderate cell death. Cell death was often preceded by axonal dystrophy, which coincided with aberrations in mitochondrial morphology and transport. The transcriptional coactivator PGC-1α but not WldS prevented both cell death and axonopathy in aSyn-expressing neurons, suggesting that regulation of mitochondrial biogenesis and ROS production might be therapeutically relevant *in vivo*.

Wild-type human aSyn has been expressed in mice (Masliah et al., 2000; van der Putten et al., 2000; Fleming et al., 2004), flies (Feany and Bender, 2000; Auluck et al., 2002) and worms (Lakso et al., 2003) in an effort to understand the relevance of this protein to PD. None of these model systems recapitulates all aspects of disease, but all have strengths that can be exploited to interrogate various aspects of aSyn toxicity (Fernagut and Chesselet, 2004; Chesselet, 2008; Lim and Ng, 2009). The limitations of the model we describe include its rapid onset, high levels of synuclein expression and confinement to peripheral sensory neurons, none of which characterize human pathophysiology in PD. However, these very limitations are also strengths of the system. Embryonic and larval zebrafish are increasingly recognized as a promising model organism for neurodegeneration research because early and robust phenotypes permit high-throughput analysis of potential therapeutic targets in a living vertebrate system (Tomasiewicz et al., 2002; Bandmann and Burton, 2010). Moreover, the optical transparency of zebrafish and the superficial location of peripheral sensory neurons present a novel method for identification and interrogation of compartment-specific degeneration pathways in aSyn toxicity.

### aSyn causes axonopathy in peripheral sensory neurons

In postmortem neurons from individuals with PD, aSyn aggregates are often observed in the axon prior to the cell body (Braak et al., 1999; Galvin et al., 1999), a feature that has also been observed in some disease models (Marui et al., 2002; Orimo et al., 2008; Schulz-Schaeffer, 2010; Volpicelli-Daley et al., 2011; Boassa et al., 2013). Early aggregation might result in early dysfunction at the presynaptic terminal, causing defects in neurotransmission long before cell death. In multiple models of PD, both toxin-induced (Herkenham et al., 1991; Orimo et al., 2008; Li et al., 2009a; Cartelli et al., 2010; Arnold et al., 2011; Kim-Han et al., 2011; Mijatovic et al., 2011) and genetic (Li et al., 2009b; Decressac et al., 2012) axon degeneration is observed prior to cell death, and in a higher percentage of cells. This has raised the question of whether PD represents a ‘dying back’ of dopaminergic neurons (Hornykiewicz, 1998), with synapse loss initiating a retrograde degenerative process that leads to cell death. We observed early axon pathology in aSyn-expressing cells, with focal swellings or widespread beading in the axon, before cell death. A higher percentage of cells exhibited axonopathy than cell death, suggesting that axon degeneration might lead to death. However, time-lapse imaging revealed that, although axonal varicosities were observed early, axonal fragmentation was not stereotyped, and did not always occur prior to death of the cell body. By contrast, after transection, WD of the distal axon proceeded with stereotyped kinetics in aSyn-expressing axons, like in wild-type cells. The early axonopathy observed in uninjured axons therefore does not cause a ‘functional’ axotomy, and the fragmentation that later occurs is not prevented by WldS. Together, these results suggest that aSyn-induced axon degeneration is not Wallerian-like. They also indicate that degeneration is not a ‘dying back’ process in which axon degeneration is required for cell death. Nevertheless, the early axonopathy could be associated with significant functional impairment, and likely represents an important therapeutic target.

Our characterization of the relationship between axonal fragmentation and cell death in this model does not rule out the possibility that independent, compartment-specific degeneration pathways are activated by aSyn. Indeed, dopaminergic neurons in JNK2/3 double-knockout mice do not die after MPTP administration, but their axons degenerate, suggesting that separate mechanisms underlie degeneration in the two compartments in a PD model (Ries et al., 2008). Likewise, WldS is protective against axon degeneration but not cell death after systemic MPTP treatment (Hasbani and O’Malley, 2006; Antenor-Dorsey and O’Malley, 2012) or application of 6-hydroxydopamine (Sajadi et al., 2004). Retrograde axonal degeneration is therefore not required for cell death in these acute models, but might benefit from independent protection. In zebrafish peripheral sensory neurons, WldS delayed the early axonopathy caused by aSyn, and had no effect on cell death, consistent with the aforementioned toxin studies. However, in WldS-expressing cells that died, axons were not preserved. Because WldS protection is dose-dependent (Mack et al., 2001), it is possible that aSyn toxicity was initiated before levels were sufficient to provide lasting protection. Future studies with inducible aSyn expression could address this question.

### Mitochondrial dysfunction and axon degeneration

Mitochondrial dysfunction might be upstream of axon degeneration in aSyn-expressing cells. At 2 dpf, we observed changes in mitochondrial density and morphology that were consistent with mitochondrial fragmentation, even in the absence of axonal dystrophy. This phenotype is consistent with recent *in vitro* studies indicating that aSyn associates directly with mitochondria, causing mitochondrial fragmentation that is associated with respiratory chain dysfunction and impaired calcium homeostasis (Chinta et al., 2010; Kamp et al., 2010; Nakamura et al., 2011; Butler et al., 2012). In mouse dopaminergic neurons, mitochondrial fragmentation causes selective degeneration of the axonal compartment, leading to motor deficits that occur before (Pham et al., 2012) or in the absence of (Lee et al., 2012) nigral cell death. It is possible, then, that aSyn increases mitochondrial fragmentation *in vivo*, impairing redox homeostasis and ATP synthesis, and thus sensitizing the axonal compartment to further insults such as mechanical injury, oxidant stress or aSyn aggregation (Gu et al., 2010).

The early mitochondrial transport deficits we observed in aSyn-expressing axons might also be pathologically relevant. Mitochondrial motility was reduced, and motile mitochondria in aSyn-expressing cells favored retrograde transport towards the cell body. Deficits in anterograde transport of mitochondria are associated with synaptic dysfunction and degeneration (Stowers et al., 2002; Weihofen et al., 2009; Misko et al., 2010; Misko et al., 2012). Mitochondrial transport deficits have been reported in the MPTP model (Cartelli et al., 2010; Kim-Han et al., 2011), and in cells expressing the PD-associated A53T mutant form of aSyn (Xie and Chung, 2012). The transport impairment we observed could therefore underlie later dysfunction. Alternatively, reduced motility could be a protective response to mitochondrial dysfunction. PINK1 and parkin orchestrate the transport arrest of depolarized mitochondria (Wang et al., 2011; Cai et al., 2012; Liu et al., 2012), which is thought to limit network impairment. The increased retrograde transport in aSyn-expressing axons could thus represent trafficking of damaged mitochondria to lysosomes in the cell body, where mitophagy is thought to occur.

A better understanding of mitochondrial dysfunction in this model could provide insight into PD pathogenesis. Many genes associated with hereditary PD converge on mitochondrial function and quality control (Cardoso, 2011; Sai et al., 2012), and both genetic and pharmacological models of PD implicate mitochondrial dysfunction in pathogenesis (Cassarino et al., 1997; Przedborski and Jackson-Lewis, 1998; Exner et al., 2012; Van Laar and Berman, 2013). In our model, focal varicosities in severely beaded axons were occupied by swollen, rounded mitochondria, similar to mice expressing a disease-associated form of human aSyn (A53T) (Martin et al., 2006; Chinta et al., 2010). Mitochondrial swelling is consistent with opening of the mitochondrial permeability transition pore (mPTP), which is sufficient to induce axon degeneration in some cell types (Barrientos et al., 2011). Opening of the mPTP is induced by calcium overload in the mitochondria (Haworth and Hunter, 1979; Gunter et al., 1994), and facilitated by ROS accumulation (Costantini et al., 1996; Kowaltowski et al., 1996; Vercesi et al., 1997). Normal pacemaking through L-type calcium channels in dopaminergic neurons causes oxidant stress and might lower the threshold for mPTP formation (Guzman et al., 2010; Surmeier et al., 2011; Goldberg et al., 2012), which could underlie the selective vulnerability of dopaminergic neurons to cell death in PD. Indeed, mitochondria isolated from the rat striatum are more sensitive to calcium influx than cortical mitochondria (Brustovetsky et al., 2003).

### PGC-1α protects against aSyn toxicity

The transcriptional coactivator PGC-1α plays crucial roles in regulating mitochondrial biogenesis and ROS scavenging, and could be a therapeutically relevant target in the treatment of neurodegenerative disease (Anderson and Prolla, 2009; Handschin, 2009; Zheng et al., 2010). Defects in PGC-1α activity were recently reported in fibroblasts from individuals with early-onset, parkin-deficient PD (Pacelli et al., 2011), and genome-wide association studies identified reduced expression of many PGC-1α-regulated genes in tissues from individuals with PD (Zheng et al., 2010). We found that overexpression of mouse PGC-1α protects against aSyn toxicity in both the axon and the cell body. Others have reported that it protects mouse dopaminergic neurons from MPTP toxicity (St-Pierre et al., 2006; Mudò et al., 2012). This effect seems to be mediated by upregulation of ROS detoxification programs, including increased expression of mitochondrial superoxide dismutase (SOD2) (St-Pierre et al., 2006). Siddiqui and colleagues recently reported that aSyn associates with PGC-1α during oxidative stress, inhibiting these protective effects; however, overexpression of PGC-1α reestablished protection (Siddiqui et al., 2012). PGC-1α and its downstream target genes might therefore be relevant therapeutic targets in the treatment of synucleinopathies (Tsunemi and La Spada, 2012).

## MATERIALS AND METHODS

### Fish

Fish were raised on a 14 hour/10 hour light/dark cycle at 28.5°C. Embryos were kept in a 28.5°C incubator. Experiments were approved by the Chancellor’s Animal Research Care Committee at the University of California, Los Angeles.

### Transgenes

A plasmid encoding aSyn and the viral T2A cDNA sequence cloned into pDsRed-Monomer N1 vector (Clontech) has been described elsewhere (Prabhudesai et al., 2012), and was cloned into the p3E entry vector of the Tol2/Gateway zebrafish kit (Kwan et al., 2007). The T2A sequence causes ribosomal ‘skipping’ (Donnelly et al., 2001), generating two proteins from a single open reading frame and resulting in stoichiometric expression of the gene of interest and the fluorescent reporter (Tang et al., 2009). The T2A-DsRed cDNA was cloned into the p3E entry vector of the Gateway system (Invitrogen), downstream of a multiple cloning site (MCS) (Kwan et al., 2007). Because GFP expression is brighter than monomeric DsRed and is therefore preferable for axon imaging, the T2A sequence was also cloned into the p3E entry vector between an MCS and GFP. aSyn was then cloned into the MCS to generate p3E-aSyn-2A-GFP. WldS or mouse PGC-1α (Hanai et al., 2007) (gift from Dr Shintaro Imamura) was inserted into the p3E-MCS-T2A-DsRed plasmid. In all constructs, the *CREST3* enhancer (gift of H. Okamoto) (Uemura et al., 2005) in the p5E entry vector drove expression of Gal4 and 14×UAS (Köster and Fraser, 2001) in pME, and these were recombined with one of the p3E donor vectors to generate the following transgenes:

A: *CREST3*:Gal4:UAS:GFP
B: *CREST3*:Gal4:UAS:aSyn-2A-GFP
C: *CREST3*:Gal4:UAS:DsRed
D: *CREST3*:Gal4:UAS:WldS-2A-DsRed
E: *CREST3*:Gal4:UAS:PGC-1α-2A-DsRed.

To visualize mitochondria, a cox8 mitochondrial targeting sequence was added to DsRed and cloned into the Gateway system to generate UAS-mitoDsRed-polyA (mitoDsRed; gift of Carla Kohler laboratory, University of California, Los Angeles, CA). This was co-injected with Plasmid A or B above so that the *CREST3* enhancer drove expression of GFP (+/− aSyn) and mitoDsRed in the same neurons. Approximately 15 pg of each transgene were injected into embryos at the one-cell stage for transient, mosaic transgene expression in sensory neurons, and embryos were screened at 1 and 2 dpf for reporter expression. Because DsRed maturation proceeds more slowly than GFP, robust expression of DsRed reporter transgenes was not observed until 2 dpf, so this was the earliest time point for all experiments.

### Immunohistochemistry

At 48 hpf embryos were dechorionated and fixed with 4% paraformaldehyde in PBS, pH 7.4, at 4°C overnight. Fixed embryos were cryoprotected with 30% sucrose and embedded into OCT Compound (Electron Microscopy Sciences) for frozen sectioning. 10-μm sections were produced using a cryostat (Leica CM3050) and bonded to glass slides. Sections were washed with PBS, blocked with 10% normal goat serum, and incubated with anti-aSyn mouse IgG primary antibody (BD Biosciences) at 1:500 dilution at 4°C in a humidified chamber overnight. Slides were again washed in PBS and incubated with Alexa-Fluor-594-conjugated goat anti-mouse IgG (Invitrogen) secondary antibody at 1:500 dilution for 2 hours at room temperature and with 4′,6′-diamidino-2-phenylindole (DAPI) for nuclear staining. Single-channel images were obtained with a fluorescence microscope (Eclipse e400, Nikon) and merged using Adobe Photoshop software.

### Imaging

Embryos were dechorionated, anesthetized in 0.01% tricaine, mounted in 1.2% low-melt agarose (Promega) in sealed chambers (O’Brien et al., 2009) and imaged on a heated stage with a 20× air objective on a confocal microscope (Zeiss LSM 510), using a 488 nm laser line for GFP and 543 nm for DsRed. Cell death was initially quantified in cells expressing only GFP or aSyn-2A-GFP The counts were also performed in embryos co-injected with a DsRed reporter transgene, to allow later comparison with WldS- and PGC-1α-expressing cells.

For time-lapse analysis of axon degeneration and cell death, embryos were imaged every 20-60 minutes for up to 12 hours. Images were compiled into projections and movies with QuickTime software.

To determine the effect of aSyn expression on mitochondrial density and morphology, mitoDsRed-expressing embryos were imaged at 2 dpf using a 40× oil objective and 3× digital zoom. Mitochondrial transport was visualized by time-lapse imaging of a single optical section using only the 543 nm laser, at a frequency of ~1 Hz, for 6 minutes.

### Axon transection

GFP- and aSyn-2A-GFP-expressing axons were cut using a Zeiss 710 microscope equipped with a multiphoton laser (O’Brien et al., 2009). Embryos were imaged with a 25× water objective and 488/543 nm laser scanning to identify the axonal region of interest, then 1-5 scans of the two-photon laser (tuned to 910 nm) were used to transect an axonal region of interest at 100× digital zoom.

### Quantification of mitochondrial morphology and transport

All axons within an image were traced using ImageJ software. Line length was calibrated to convert pixels to distance, and the Measure plugin was used to quantify total axon length. Density was calculated as mitochondria/axon length. Mitochondrial morphology was calculated as the ratio of length to width; all mitochondria within an image were quantified. Mitochondrial motility was defined as the percent of total mitochondria that moved in a 50-μm axon segment during a 6-minute movie, and was quantified using the Kymograph macro for ImageJ. A mitochondrion was considered to be moving only if it traveled at least 2 μm at a speed of at least 0.1 μm/s (Misgeld et al., 2007). Speed was calculated as the slope of distance (*x*) over time (*y*, in pixels) on the kymograph, and direction was determined by the sign of the slope. Mitochondrial transport behaviors were characterized by quantifying the percentage of time that motile mitochondria spent paused or moving in the anterograde or retrograde direction.

### Data analysis

Data were analyzed with GraphPad Prism software. Unpaired *t*-tests were used to evaluate changes in mitochondrial morphology and transport between WT and aSyn-expressing cells, and to quantify cell death in GFP-and aSyn-2A-GFP-expressing cells. Minimal significance was set at *P*<0.05. One-way ANOVA and planned, unpaired Student’s *t*-tests were used to evaluate the effect of aSyn on cell death and axon degeneration, and the ability of PGC-1α or WldS to prevent those effects. One-way ANOVA was followed by the appropriate post-test to correct for multiple comparisons.

## Supplementary Material

Supplementary Material
