# Supplementary material for: Axon degeneration and PGC-1α-mediated protection in a zebrafish model of α-synuclein toxicity
Source: Dis Model Mech. 2014 Mar 13;7(5):571–82. doi: 10.1242/dmm.013185 (PMC4007408; doi:10.1242/dmm.013185)
Supplement: Supplementary Material [file supp_7_5_571__index.html]

Axon degeneration and PGC-1α-mediated protection in a zebrafish model of α-synuclein toxicity — Supplementary Material 

# Axon degeneration and PGC-1α-mediated protection in a zebrafish model of α-synuclein toxicity

## DMM013185 Supplementary Material

**Files in this Data Supplement:**

- **Supplementary Material**
