# Supplementary material for: Axon degeneration and PGC-1α-mediated protection in a zebrafish model of α-synuclein toxicity
Source: Dis Model Mech. 2014 Mar 13;7(5):571–82. doi: 10.1242/dmm.013185 (PMC4007408; doi:10.1242/dmm.013185)
Supplement: Supplementary Material [file supp_7.5.571_DMM013185.pdf]

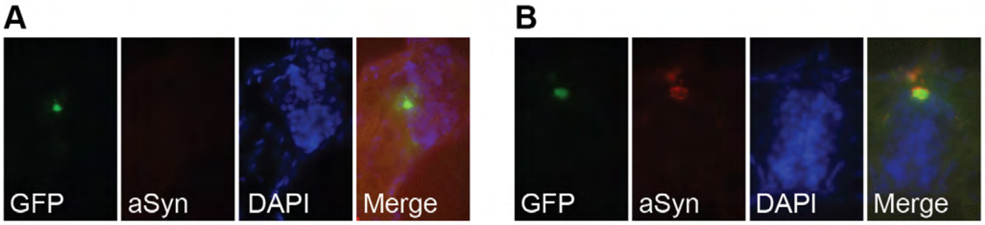

Supplementary Fig. S1. Alpha-synuclein aggregates in zebrafish peripheral sensory neurons. Embryos were injected at the 1-cell stage with transgenes indicated in Fig. 1A. At 48 hours post-fertilization, embryos were fixed in 4% PFA, sectioned, and stained with anti-alpha-synuclein primary antibody, followed by Alexa 594-conjugated goat anti-mouse IgG secondary antibody. DAPI staining was used to visualize nuclei. (A,B) Cross-sections through the spinal cord of GFP- or aSyn-2A-GFP-expressing embryos. Green fluorescence indicates Rohon-Beard cells expressing the transgene. No aSyn staining was observed in control-injected cells (A). All aSyn-injected cells exhibited red aSyn staining (B).

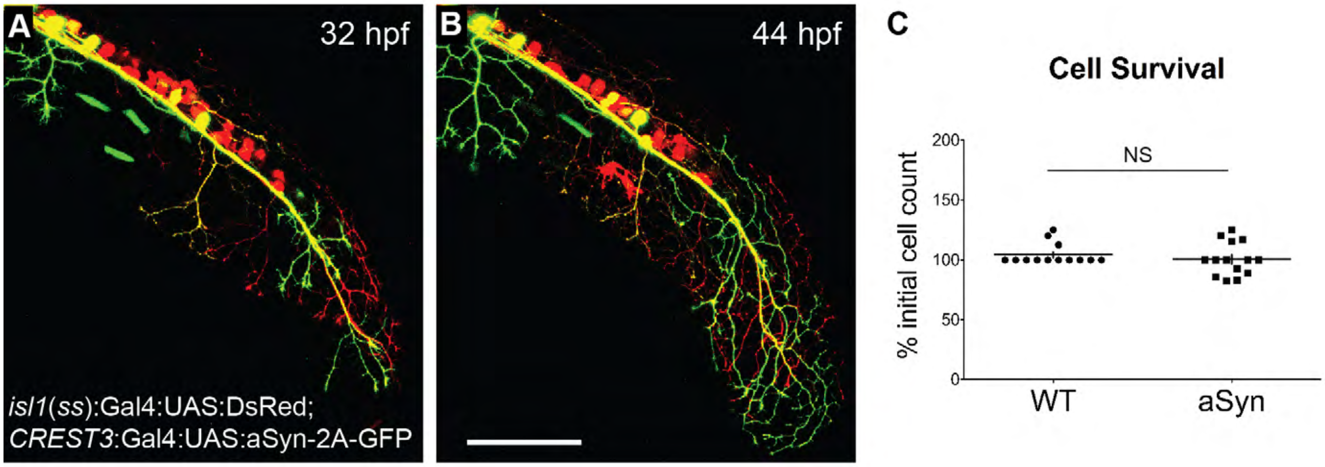

Supplementary Fig. S2. Alpha-synuclein expression does not impair early survival or axonal outgrowth of peripheral sensory neurons. The *CREST3*:Gal4:UAS:aSyn-2A-GFP transgene was injected into embryos from a previously described stable line (Palanca et al., 2013) expressing DsRed in peripheral sensory neurons (*isl1(ss)*:Gal4-UAS-DsRed). Embryos were imaged every hour between 32 (A) and 44 (B) hours post-fertilization (hpf). Axons grew normally during this time. (C) Cell bodies were counted, and cell survival at 44 hpf was quantified as a percentage of the earlier time point. There was no difference in survival between WT and aSyn-expressing cells by 44 hpf (WT: 104.4±2.4% aSyn: 100.7±3.7%; $n \geq 13$ embryos; $P=0.4203$).

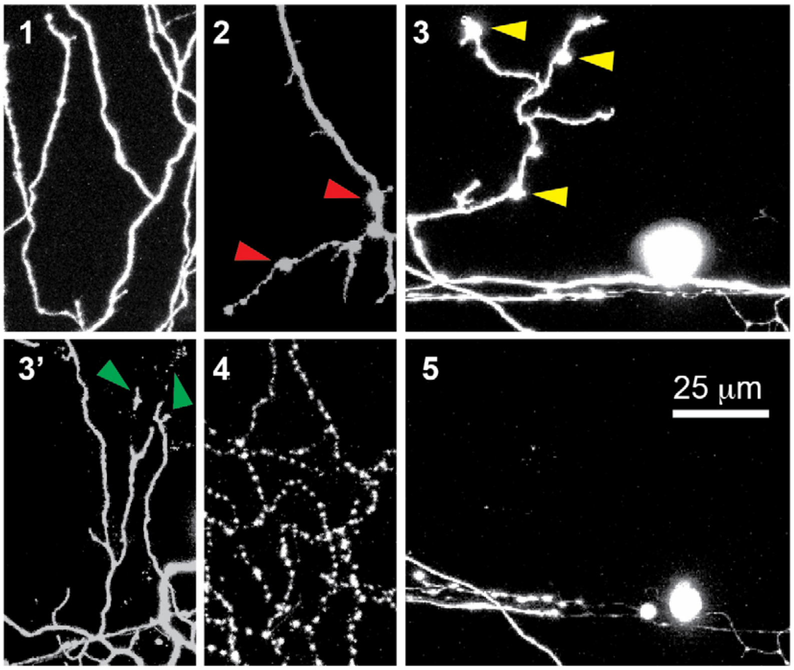

1	Smooth, continuous axons
2	Mild beading (diffuse or locally restricted)
3	Severe beading, +/- limited fragmentation
4	Diffuse fragmentation
5	Most/all fragments cleared

Supplementary Fig. S3. Axon degeneration index. (A) five-point scoring system was devised to quantify axon morphology between 2 and 3 dpf. A score of 1 was given to smooth, continuous axons. A score of 2 was given to axons with mild and diffuse or moderate but localized swellings. A score of 3 was given to axons that either had severe swelling or a small amount of fragmentation (as seen at the tips in 3′). Axons with more extensive fragmentation were given a score of 4. When fragments were mostly or entirely cleared, axons received a score of 5.
